# An Empirical Study on the Evaluation of Emotional Complexity in Daily Life

**DOI:** 10.3389/fpsyg.2022.839133

**Published:** 2022-03-03

**Authors:** Boshi Dong, Guangxing Xu

**Affiliations:** ^1^The First People’s Hospital of Lianyungang, Lianyungang, China; ^2^School of Psychology and Cognitive Science, East China Normal University, Shanghai, China

**Keywords:** emotional complexity, emotional events, positive affect, negative affect, psychology

## Abstract

Emotional complexity means diversity, universality, and differentiation of individual emotions. This research consisted of two studies to demonstrate the constitution of the emotional complexity. In Study 1, the participants were asked to use 10 emotional words to record the variation of emotions over 30 days in daily life. In Study 2, the experimental materials were enriched. The participants were required to note the emotions with the Positive and Negative Affect Schedule in a 3-day holiday—all the individuals in the two studies needed to record the most important emotional event. As a result, the youth experienced complex emotions every day. Emotional complexity indicators included covariation index (*r*), component index (*C*_pc_ and *C*_unshared_), granularity index (*G*_e_, *G*_p_, and *G*_n_), and variability index (*V*_p_ and *V*_n_). A four-factor model reflected a good model fit, with 𝜒^2^/df = 0.33, CFI = 1.00, TLI = 1.03, RMSEA = 0.000 (0.00, 0.20), SRMR = 0.003, including positive differentiation, covariation of positive affect and negative affect, negative differentiation, and emotional variation. These indicators may reflect the complex experiences in everyday life. The results shed light on the emotional experience that can change greatly within 1 day and on episodes of emotional disruption resulting from an important event coupled with excessive excitement or extreme tension.

## Introduction

The complex emotions can be called “emotional complexity,” which means diversity, universality, and differentiation of individual emotions (Ben-Ze’ev and Revhon, 2004; Grossmann et al., 2016). Wessman and Ricks (1966) used “affect complexity” to describe the complications of emotional experiences. In Handbook of Emotions, Lewis et al. (2008) further advanced the concept of emotional complexity by focusing on three main aspects, including (1) dialecticism and precision in recording emotions; (2) emotional, propositional knowledge in different situations; and (3) complexity of self-characterizations.

There are also two kinds of perspectives that define emotions’ nature (Hay and Diehl, 2011; Grossmann et al., 2016). The instinctive orientation considers a series of emotions as genetic and fixed categories (Roseman, 1984; Lindquist et al., 2006). Barrett and Bliss-Moreau (2009) suggests that when more than one kind of emotion is triggered at a particular moment, or when people accurately describe the variety in their emotional experience, they will self-report the emotional complexity. Then people gain propositional knowledge about emotions when they learn to associate a given emotion with the environment. Therefore, the emotional strategy is a perceptual strategy, which means one can get the coding strategies at birth and gradually adapt to different environments (Grossmann et al., 2016). Self-characterization of emotional complexity is reflected in individuals’ ability to recognize their experiences as complex (Berrios, 2019).

Another way to define the nature of emotion uses a “psychological construction” orientation (Russell, 2003, 2017; Barrett, 2004; Feldman et al., 2007). It holds that emotional complexity is intrinsic to the emotional experiences of neurobiological and psychological systems. A discrete emotion emerges in consciousness when a more basic core affect is automatic and implicit. The core effect is a constantly changing state with valence and arousal-based properties (Russell, 2003; Barrett, 2004). Current studies use the “emotional conception system” to classify core effects, such as anger, sadness, and fear. This term refers to the knowledge about specific emotions that people understand to clarify effectively and automatically attribute core affect to a certain state that is efficient and conceptual when inner feelings of the body and the external information from the environment are bound together for a moment in time. Categorizing emotions transforms core affect into an intentional state, allowing individuals to explore what caused the emotional change, what to do next, and how to communicate to others effectively and efficiently (Barrett, 2004). Therefore, emotional complexity is the direct result of the emotional conception system.

In addition, emotional complexity includes at least two different simultaneous or sequential emotions simultaneously. A sequential or continuous state is a rapid change in two or more emotional states, such as a pure emotion appearing first and then being quickly replaced by another emotion. Conversely, a simultaneous complex emotion` responds to stimuli with two or more opposite emotions’ (Larsen et al., 2001). Rothman et al. (2017) point out that simultaneous states include mixed emotions and emotional ambivalence, and sequential states include affective transition and emotional inconsistencies. The definitions and differences in these states stem from trait factors, including effective spin, affective variability, emotional inertia, and emotional complexity (Kang and Shaver, 2004). Therefore, some current studies measure emotional complexity by the level of emotional awareness (Ben-Ze’ev and Revhon, 2004; Berrios, 2019). Some researchers stated mixed emotions as indicators of emotional complexity (Trudel-Fitzgerald et al., 2017).

Emotional complexity is multiply defined, often regarding the co-occurrence and differentiation of positive and negative emotions as the leading indices (Hay and Diehl, 2011; Grühn et al., 2013; Larsen, 2017). The co-occurrence index reflects the two opposite valences in emotions (e.g., happiness and sadness) simultaneously (Ong and Bergeman, 2004). In contrast, differentiation of positive and negative emotions, named granularity index, reflects the characteristic distinction among emotions that one makes (Ong and Bergeman, 2004).

Although most emotional experiences can be represented by positive and negative dimensions (Barrett, 1998; Charles et al., 2017; Butler et al., 2018), the degree to which adults distinguish emotions is different (Ong et al., 2017). Barrett (2004) argued that people first experience core affect, which they then categorize as a particular emotion (e.g., anger, frustration, etc.). It is essential to assess emotional complexity through the co-occurrence and granularity index. Barrett (2004) studies shows that self-reports of emotions and emotion labels used in conception reflect more forms in the emotional words when differentiating; and they also reflect the differences in adults’ phenomenological experience.

Zautra et al. (2005) proposed Dynamic Model of Affect (DMA) to explore individuals’ differences of emotions in time. DMA asserts that it is uncertain to descript emotional experiences, and pressure in situations will increase the uncertainty. Information processing requires cognitive strategies and becomes more complex when complicated emotional experiences. Consequently, DMA suggests that emotions will polarize in a high-pressure environment. For example, a high level of negative emotion is often accompanied by a low level of positive emotion. DMA aims to assess emotional states without ignoring one’s stress level, which means that the relationship between positive and negative emotion may change with information processing (such as cognitive and emotional processing methods). Moreover, it is important to construct at least two dimensions to generally classify emotions, one to assess negative emotion with the accompanying motivation level, and the other to assess the level of positive emotion with the accompanying processing (Ramsey et al., 2016).

One groundbreaking approach uses “micro longitudinal” techniques to assess emotions and intense events at multiple points (Kreibig and Gross, 2017; Ong et al., 2017). This method involves noting the correlation between positive and negative emotions at each time point and observing what happens to this association when pressure or other destructive events occur in daily life. Furthermore, people can determine whether a stressful event was greater in magnitude or occurred with greater frequency than their average experience by repeatedly measuring these co-occurring experiences. To obtain the deviations from the mean values, the independent variables are person-centered. Person-centered negative affect (NA) can be used as a predictor in a typical model to estimate its association with positive affect (PA) over time in the context of a linear mixed model. In this case, choosing which valence should be modeled as the independent versus dependent variable is random but may be guided by particular research questions.

This study aimed to explain daily emotional experience traits in the youth and considered emotional complexity as a state (the co-occurrence of positive and negative emotions). To investigate the simultaneous and sequential emotional states, we set up two studies to explore the indicators to describe the complex emotions in everyday life. Through the studies, we could find the emotional complexity index and investigate the relationship among the indicators. And also, we would examine the emotional states of the daily experiences in a different environment.

## Materials and Methods

Study 1 contained 61 participants without psychiatric disorders from a normal university in East China who were initially invited to join in the study. All the participants were drawn randomly from the university. Fifteen participants who completed less than half of the planned measurement points (once a day for 30 days) were excluded. The final sample included solely Han Chinese, ranging in age from 18 to 24 years old; most of the sample were women (53%). Participants signed an informed consent document before recording the emotions. After 30 day-dairy, participants were rewarded for a gift.

Study 2 consisted of 46 participants recruited from a normal university in East China without psychiatric disorders. This sample included solely Han Chinese, ranging in age from 18 to 24 years; most of the sample were women (70%). Before initiating the experiment, participants read and signed an informed consent document. After completing the experiment, participants were rewarded with a gift.

### Procedures

Study 1: Using an online questionnaire, participants were asked how often (1 = none, 7 = always) they felt each emotion in the last 24 h (Ready et al., 2007, 2008). The emotions consisted of positive and negative items, including happy, joyful, pleased, enjoyment/fun, content, depressed, unhappy, frustrated, angry/hostile, and worried/anxious. Participants completed the online questionnaire about their emotions and experiences for 30 consecutive evenings.

Study 2: Data were collected during the three-day New Year holiday using an affect measure with more independent PA and NA scales than the instrument used in study 1. Demographic information was entered online, and the Positive and Negative Affect Schedule (PANAS) was completed online every day over the 3-day holiday (Watson et al., 1988). The PANAS exhibited strong internal consistency (PA *α* = 0.90; NA *α* = 0.87) and 2-month test–retest reliability (PA *r* = 0.47; NA *r* = 0.39).

### Data Analysis

Consistent with existing research, this study used principal components analysis (PCA) to estimate the data of emotional records in study 1 and study 2 (Carstensen et al., 2000; Ong and Bergeman, 2004; Grühn et al., 2013). The covariation score was defined as the within-person correlation between daily reports of positive and negative (*r*_pn_), where a coefficient close to 0 indicated greater complexity among emotions. The component scores were obtained by within-person principal components factor analysis. They were taken from the number of factors with eigenvalues greater than 1, where higher values indicated higher levels of emotional complexity. The other component score was calculated by subtracting the variance accounted for in the first extracted principal component factor from 100% (*C*_unshared_).

Granularity scores were computed from each participant’s inter-class correlation coefficient (ICC) index subtracted from one (McGraw and Wong, 1996; Grühn et al., 2013). Low granularity scores reflect high consistency among emotion reports and different forms of emotion expressions. Therefore, we calculated three items: emotion granularity (*G*_e_), positive emotions (*G*_p_), and negative emotions (*G*_n_). The variability scores consisted of two items calculated from the standard deviation of the positive and negative emotions from the experience-sampling data, including positive affect (*V*_p_) and negative affect (*V*_n_).

Statistical analysis was conducted with SPSS 25.0 and Mplus 7.4. The data from participants who reported the same score continuously in the records were excluded from the analysis. The participants need to report the most important emotional events in the two studies. The emotional reaction to the event could be strong and last all day. Then the narratives were coded for numbers of emotional units (i.e., sentence/phrase that conveyed a complete thought). Narratives also were coded for thematic content: Physical health; Interpersonal social; Recreation; Education; and Natural environment. The discrepancies of all coding were resolved *via* discussion.

## Results

Table 1 showed that the daily life PA and NA scores were close to the emotional scores in holiday. For instance, PA decreased, and NA scores were lower than baseline because of the environment change and the effect of time. The effects also involved emotional vocabulary and cultural background (Bagozzi et al., 1999). The vocabulary selected in Study 2(a) reflected the core affect, and the PANAS responses in Study 2(b) reflected the emotional survey, especially regarding the polarization of emotions. However, in Study 1, the emotional adjectives were based on the Chinese context for special emotions. At the same time, Study 2 resulted in different environments, consistent with the conclusion in Study 1.

**Table 1:** Descriptive statistics of the emotional records in study 1.

	*M* ± SD	Frequency (%)
PA	4.25 ± 1.74	30.82
NA	2.09 ± 1.25	2.90
PA and NA		65.80
None		0.47
Correlation	−0.41 ± 0.28	

*PA, positive affect; NA, negative affect; None: “1” means no such emotion in the emotional report*.

**Figure 1:**
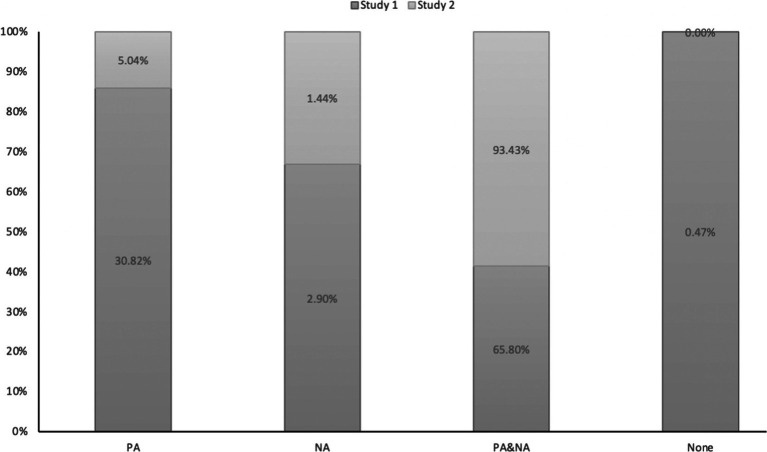
Frequency of positive affect and negative affect in Studies 1 and 2.

The within-person correlation between PA and NA in Study 1 was moderately negative (see Table 1). However, in Study 2, there was no correlation between PA and NA (see Table 2). The emotional frequency of individuals’ experiences showed the same trend in the two experiments. Participants reported more positive emotions than negative emotions in daily life and over the holiday. The proportion of PA and NA occurring simultaneously was the highest in the two experiments. The no-emotion proportion was only 0.47% among the four emotional reports, especially in Study 2. And the participants did not report any emotions on holiday. It was noteworthy that the total proportion of no-emotion and NA reports accounted for only 1.44% of the variance, whereas PA and NA accounted for 93.43% (see Table 2 and Figure 1). The results indicated that the youth’s emotional experience was richer and more diverse during the holidays.

**Table 2:** Descriptive statistics of the emotional records in study 2.

	*M* ± SD	Frequency (%)
PA	2.91 ± 0.82	5.04
NA	1.99 ± 0.76	1.44
PA and NA		93.43
None		0
Correlation	0.01 ± 0.8	

*PA, positive affect; NA, negative affect; None: “1” means no such emotion in the emotional report*.

Table 3 showed the indicators of emotional complexity, including the covariation index (r); component index (*C*_pc_ and *C*_unshared_); granularity index (*G*_e_, *G*_p_, and *G*_n_) and variability index (*V*_p_ and *V*_n_). There were different degrees of correlation between the indicators. The covariation score had a significant correlation with the component and granularity scores, except for Cunshared and the variability scores. However, there was a lack of component indicators in Study 2 (see Table 4). The results also showed that *G*_n_ was positively correlated with *V*_p_, which differed from the results in Study 2 (Table 5).

**Table 3:** Associations between indicators of emotional complexity in Study 1.

	*r*_pn_	*C*_PC_	*C*_unshared_	*G*_e_	*G*_p_	*G*_n_	*V*_p_	*V*_n_
*r*_pn_	1							
*C*_PC_	0.59^**^	1						
*C*_unshared_	0.16	0.11	1					
*G*_e_	−0.74^**^	−0.43^**^	0.10	1				
*G*_p_	0.33^*^	0.42^**^	0.59^**^	0.04	1			
*G*_n_	0.60^**^	0.66^**^	0.44^**^	−0.28	0.48^**^	1		
*V*_p_	−0.12	−0.26	−0.38^*^	−0.15	−0.65^**^	−0.07	1	
*V*_n_	−0.29	−0.53^**^	−0.31^*^	0.13	−0.48^**^	−0.52^**^	0.54^**^	1

*r*_pn_, covariation score; *C*_pc_ and *C*_unshared_, component score; *G*_e_, *G*_p_, and *G*_n_, granularity score; *V*_p_ and *V*_n_, variability score.

* *p* < 0.05

** *p* < 0.01.

**Table 4:** Associations between indicators of emotional complexity in Study 2.

	*r*_pn_	*G*_e_	*G*_p_	*G*_n_	*V*_p_	*V*_n_
*r*_pn_	1					
*G*_e_	−0.445^**^	1				
*G*_p_	0.096	0.183	1			
*G*_n_	−0.122	−0.035	−0.038	1		
*V*_p_	0.113	−0.124	−0.507^**^	0.418^*^	1	
*V*_n_	0.035	−0.127	−0.381^**^	−0.517^**^	0.116	1

*Correlation is significant at the 0.05 level (*p* < 0.05). **Correlation is significant at the 0.01 level (*p* < 0.01).

**Table 5:** Latent factors.

	PD	COV	ND	EV
*r*	0.70	0.39		
*C*_PC_	0.94			0.29
*C*_unshared_	−0.95			
*G*_e_		0.97		
*G*_p_	−0.99		0.39	
*G*_n_			−0.77	0.66
*V*_p_	0.90		0.04	0.53
*V*_n_		−0.44	0.577	

*PD, positive differentiation; COV, covariation of PA and NA; ND, negative differentiation; EV, emotional variation*.

**Figure 2:**
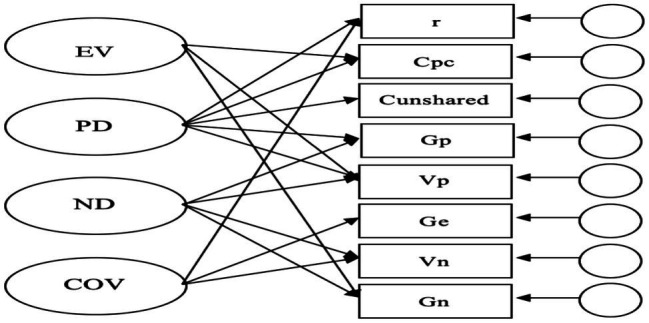
Confirmatory factor analysis on indicators of emotional complexity.

To further identify the predictive effects of the indicators for emotional complexity, we performed Exploratory Factor Analysis and Confirmatory Factor Analysis (Hopwood and Donnellan, 2010; Khalil et al., 2021; Khawaja et al., 2021). A four-factor model reflected a good model fit, with 𝜒^2^/df = 0.33, CFI = 1.00, TLI = 1.03, RMSEA = 0.000 (0.00, 0.20), SRMR = 0.003. The results showed that some indicators were related to multiple factors (see Table 4 and Figure 2).

### Important Emotional Events

The period from 18 to 24 years old in China is filled with education; just as the participants’ records of important events, the most frequent emotional event was “courses.” Because the participants were mainly selected from normal universities, life in the campus was closely related to their daily experiences. However, the courses included calligraphy, piano, painting, dancing, and professional lessons. As a result, the participants could feel more complex emotions, such as depression, excitement, sadness, anxiety, calm, happiness, etc. The narrative could also be described by the words “*busy and productive*” (Butler et al., 2018; Sarfraz et al., 2019; Gull et al., 2021; Xiaolong et al., 2021), not only because most of the participants were first-year students but also the heavy professional courses and colorful activities occupied the campus time with no leisure. Then life was dominated by complex emotions.

In the participants’ logs, the negative emotions, such as fatigue, irritability, and discomfort, were often associated with physical health, including “feeling cold,” “illness,” and “discomfort” (see Table 6). While emotions related to “homesickness” were depression and anxiety. “Examination” was also an important cause of negative emotions, such as tension and fatigue before the exam and concerns about performance. The other topics that mostly resulted in the mood swings were weather, diner, and journey. The events for the first time in individuals’ experiences affected the occurrence and development of emotions, including intimate relationships and part-time work.

**Table 6:** Frequency of important emotional events.

Important emotional events		Frequency	
		Positive affect	Negative affect
Life in campus	Courses	119	69
	Homework (calligraphy, piano, painting, dancing, etc.)	96	85
	Examination	45	62
	Activities on campus (meetings, competitions, dinners, etc.)	78	69
	Intimate relationship	27	25
	Dormitory relationships	10	30
	Daily life (getting up, sleeping, eating, etc.)	55	60
Natural environment	Weather (sunny, rainy, windy, etc.)	15	20
Personal factors	Physical health (feeling cold, illness, discomfort, etc.)	0	32
	Introspection	40	46
Entertainment	Online games and mobile games	34	30
	Sports (running, basketball, volleyball, etc.)	58	16
	Communication with family members	76	12
Extracurricular activities	Practice, part-time work	20	20
	(Journey, home)	89	78

At the same time, attention to social events made participants experience complex emotions. Typical events included: “I was satisfied with the state of learning, but I felt depressed and distressed for the victims in the nanny;” “Hell is empty and demons are on earth. The arson case that has been concerned has not been concluded so far. However, the other cases ended up with nothing definite. In fact, the case named three colors have been disclosed for 15 years, but it was not until these two days those social concerns began.”

## Discussion

The PA and NA scores in Study 2 were lower than those in Study 1; when the emotional record was short and away from the familiar environment, the PA and NA scores changed substantially. This may be explained by errors in statistical analysis or the relationships among emotional complexity, time, and environmental cues. Regarding the correlation between PA and NA, the 30-day records showed significance, but the 3-day records were insignificant, and the related coefficient was close to zero. Results in Study 1 demonstrated that while some people experienced many different emotions, their positive and negative emotions tended to be opposite (Reich et al., 2003; Grühn et al., 2013; Scott et al., 2014). There was no correlation between PA and NA in Study 1, indicating that these emotional records were discrete, or that individuals experienced a deeper complexity of emotions when separated from their usual environment. Individuals were more likely to experience the excitement on graduation and leisure days, which would reduce pressure and tension (Larsen et al., 2001).

The results of emotional frequency were consistent with previous studies (Grühn et al., 2013), but the proportion of no-emotion in Study 1 was 0, and PA and NA accounted for more than 90% of the variance. This extreme result suggests that when individuals interrupted their habits, their ability to distinguish emotions decreased, and some experienced emotional ups and downs during the exceptional time, which might affect the occurrence of PA and NA. In other words, richer emotional events resulted in more complex emotions. This suggests the co-activation of PA and NA; when the emotional valence was different, a complex emotional experience occurred.

The emotional complexity indicators in studies were inconsistent because Study 2 only collected data for 3 days, resulting in a smaller amount of data, which could not be analyzed with factor analysis. This made it difficult to get the component indicators of *C*_pc_ and *C*_unshared_. There was no fluctuation indicator in present studies, which differed from the results of previous studies (Grühn et al., 2013). The potential factors obtained by confirmatory factor analysis were consistent with the former study. Still, the items included in the factors were different because the emotions in studies were based on days, and the data in the formal study were based on time points. Different approaches to data processing also led to differences between previous results and present consequences.

The covariation index represented the overall trend of change, where PA and NA were moderately negatively correlated. The low covariation between PA and NA suggested a bipolar tendency of emotions, suggesting that individuals may experience a higher degree of emotional complexity. The high correlation between PA and NA indicated that individuals could not recognize emotional states. The low correlation indicated the relative independence of PA and NA, and it may suggest that people experience complex emotions more easily (Palgi et al., 2014). The covariation between PA and NA was more negative under non-stress conditions where the correlation coefficient was almost zero (Scott et al., 2014). The component index resulted from factor analysis, which can only calculate an index when the data reach a certain statistic. For example, Study 2 was limited to only 3 days, and insufficient data were obtained to conduct a factor analysis.

Granularity indicators were quantified by the individual’s ability to emphasize the value and arousal of core affect when reporting experiences. High scores were derived from using different vocabulary to describe diverse experiences, such as anger and sadness, which were completely different emotional experiences. Participants with low emotional granularity used the same series of words to describe the experience as participants with high emotional granularity. Still, the low granularity participants used the words to express basic emotional states. They used “anger,” “sadness,” or “fear” to describe “unhappy;” and “excited,” “delight,” or “cool” to describe “happy.” There were also a few who reported “excited” and “nervous” as similar experiences, and they replaced these words with similar vocabulary in arousal. Low granularity reflected a high degree of consistency in the emotional reports, whereas high granularity indicated that individuals used different emotional vocabulary to describe their experiences. Participants with high granularity reported different emotions over a given time, suggesting a superior ability to distinguish emotions. In contrast, participants with low granularity only reported one kind of emotion.

The variability indicator showed a tendency toward emotions, but there was no significant correlation with mental health, happiness, or personality (Grühn et al., 2013). The results also demonstrated that the covariation index, component indicators, and variability indicators were not related to mental health or alexithymia. This might be explained by a cognitive development perspective (Labouvie-Vief et al., 2007), where emotions involve both complexity and different levels of integration, suggesting a meaningful combination of experiences concerning self and others. In this view, it may be necessary to clarify whether complexity was incorporated into adaptive adjustment. Labouvie-Vief and Medler (2002) conducted a series of surveys and found that integrated individuals were highly complex and integrated. Complex individuals had high complexity and low integration. The complexity of defensive individuals was low, and the degree of integration was high, while the dysfunctional individuals were low in complexity and integration. These results suggest that complexity could not reflect the function of adaptation, but the complexity is related to dysfunction.

### Limitations and Future Research

Emotional complexity is an intricate conception. The purpose of the present studies was to evaluate the youth’s emotional complexity and explore different realistic and theoretical issues. However, there were some limitations that the data pertaining to the same event can point to very different implications about emotional complexity. For example, depending on whether data were gathered *via* a rating task or *via* diary data averaged over days. Therefore, the definition and measurement of emotional complexity is important to adult development because there

is a risk of mixed and confusing signals from emotional complexity research when different metrics are compared.

Measurement issues also matter if one is curious about potential correlates of emotional complexity. Emotion measures with subscales or items that are relatively independent may be ideal. Another issue to tackle is whether particular emotional complexity metrics have similar meanings. Future researchers should pay close attention to methodology to address these concerns. This study presented data from diary studies in daily life. It tested several theory-based predictions using numerous conceptualizations of complex emotions. Given the novel conclusions based on the data and the lack of long-term follow-up, it is impossible to determine whether co-occurrence is adaptive for later outcomes. Future studies should consider these procedures and findings within a longitudinal framework. In addition, small convenience samples were used.

## Conclusion

In summary, the current studies provide a systematic examination of the experience of complex emotions in daily life among the youth. The results shed light on the emotional experience that can change greatly within 1 day and on episodes of emotional disruption resulting from an important event coupled with excessive excitement or extreme tension. These results are fully consistent with the definition of emotional complexity, which asserts that people can experience two or more emotions simultaneously or sequentially. The results also fit well with the primary indicators of emotional complexity, PA and NA, which coexist in the individual’s experience where pure emotion rarely appears.

## Data Availability Statement

The raw data supporting the conclusions of this article will be made available by the authors, without undue reservation.

## Ethics Statement

Ethical review and approval was not required for the study on human participants in accordance with the local legislation and institutional requirements. The patients/participants provided their written informed consent to participate in this study.

## Author Contributions

All authors listed have made a substantial, direct, and intellectual contribution to the work and approved it for publication.

## Conflict of Interest

The authors declare that the research was conducted in the absence of any commercial or financial relationships that could be construed as a potential conflict of interest.

## Publisher’s Note

All claims expressed in this article are solely those of the authors and do not necessarily represent those of their affiliated organizations, or those of the publisher, the editors and the reviewers. Any product that may be evaluated in this article, or claim that may be made by its manufacturer, is not guaranteed or endorsed by the publisher.
